# Purely extradural thoracal Schwanoma: Case report

**DOI:** 10.1016/j.amsu.2022.104306

**Published:** 2022-08-05

**Authors:** Rohadi Muhammad Rosyidi, Dewa Putu Wisnu Wardhana, Wahyudi, Bambang Priyanto, Kevin Gunawan

**Affiliations:** a Department of Neurosurgery, Medical Faculty of Mataram University, West Nusa Tenggara General Hospital, Mataram, Indonesia; b Department of Neurosurgery, Udayana University Hospital, Medical Faculty of Udayana University, Bali, Indonesia; c Faculty of Medicine, Muhammadiyah Makassar University/Department of Neurosurgery, Medical Faculty of Hasanuddin University, Makassar, Indonesia; d Department of Neurosurgery, Medical Faculty of Indonesia University, Cipto Mangunkusumo National General Hospital, Jakarta, Indonesia

## Abstract

**Introduction:**

and importance. Schwannomas are benign tumors that often occur in the medulla spinalis. It arises from the Schwann cells which form the sheath of peripheral nerves. Schwannomas comprise about 30% of primary intraspinal neoplasms and approximately 75% in intradural, 10% intra-extradural, and 15% extradural. Purely extradural schwannoma is rare.

**Case presentation:**

We present two adult males with extradural thoracal schwannoma. In this case, the main complaint is paraparesis and followed by suffered low back pain and spastic. The initial symptoms of this tumor depend on the level of the tumor location. Usually begins with localized pain, sharp and transient, followed by radicular pain and radiculopathy. We reported cases of schwannoma of the thoracal spine that have presented with neurological involvement. As a result of contrast, an MRI of the spine showed tumor extradural mass lesion extending from thoracal 1–2 vertebral and thoracal 4–6 vertebral level.

**Clinical discussion:**

The location of this tumor is rare because located in the extradural (15%), which are present between the bone structure and the dura. Surgery is the treatment of choice, in this case, usually results from excellent prognostic. This patient underwent hemilaminectomy and complete surgical resection.

**Conclusion:**

Early diagnostic and complete surgical resection before the occurrence of severe symptoms will show an excellent prognosis.

## Highlights

• Purely extradural schwannoma is rare.
• We presented a schwannoma of the thoracal spine that have presented with neurological involvement.
• Early diagnostic and complete surgical resection before the occurrence of severe symptoms will show an excellent prognosis.

## 1 Introduction

Schwannomas are benign tumors that often occur in the medulla spinalis [1]. It arises from the Schwann cells which form the sheath of peripheral nerves [2]. Schwannomas comprise about 30% of primary intraspinal neoplasms and approximately 75% in intradural, 10% intra-extradural, and 15% extradural. Purely extradural schwannoma is rare [3,4]. Schwannomas most commonly occur in the age group 40–60 years and there is no difference between males and females [5,6]. The incidence is around 0.3–0.4 cases/100.000 persons per year [7]. Clinical presentation of schwannomas is endured pain, spinal root deficits, pyramidal tract compression, and sphincter disorders [8]. Gold standard treatment for symptomatic spinal schwannomas is complete surgical resection, which stops symptoms progression, helps recovery in most patients, and decreases the rate of recurrence [9].

## 2 Case report

We report 2 cases of patients with extradural thoracal schwannoma.

### 2.1 Patients 1

The first case is 60 years old male who presented to us with paraparesis and spastic for more than 3 months. The patient also had low back pain. He had no abnormality of bladder dan bowel habits. His past medical history and family history were insignificant. On examination of the lower extremity, there was paraparesis with a power of 3/5, increased physiological reflex, and positive Babinski pathological reflex. No sensory level defect was detected and no sign of spinal deformity.

**Fig. 1:**
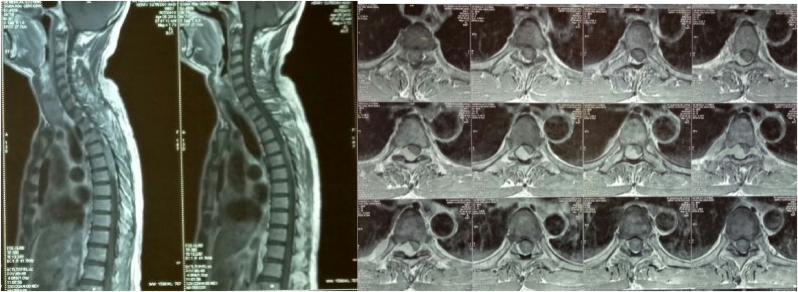
Magnetic Resonance (MR) images (T1 Sagital and Axial Section with contrast) of the spine show an extradural mass in Th4–Th6. This image shows a heterogeneous thoracal mass corresponding to a schwannoma. Magnetic Resonance (MR) images of the Th4–Th6 spine show the tumor compressing the thoracal cord.

His laboratory and thorax X-ray showed no apparent abnormalities as well. Magnetic Resonance Imaging (MRI) revealed findings of benign soft tissue consisting of proliferating cells with a spindle shape partly with hypercellular areas and partly with hypocellular areas. The conclusion was that the tissue was a schwannoma tumor. (Fig. 1).

### 2.2 Patients 2

A Male 20 years old was admitted to the neurosurgery department with a main complaint of paraparesis 6 months ago. The patient also suffered low back pain and spastic. He had no abnormality of bladder dan bowel habits. His past medical history and family history were insignificant. His physical examination was normal. Neurological examination reveals paraparesis with the power of 2/5 in both lower limbs, hypertonia in bilateral lower limbs, and increased tendon reflexes in the lower limbs. Babinski reflexes were positive in bilateral lower limbs. No sensory level defect was detected and no sign of spinal deformity.

**Fig. 2:**
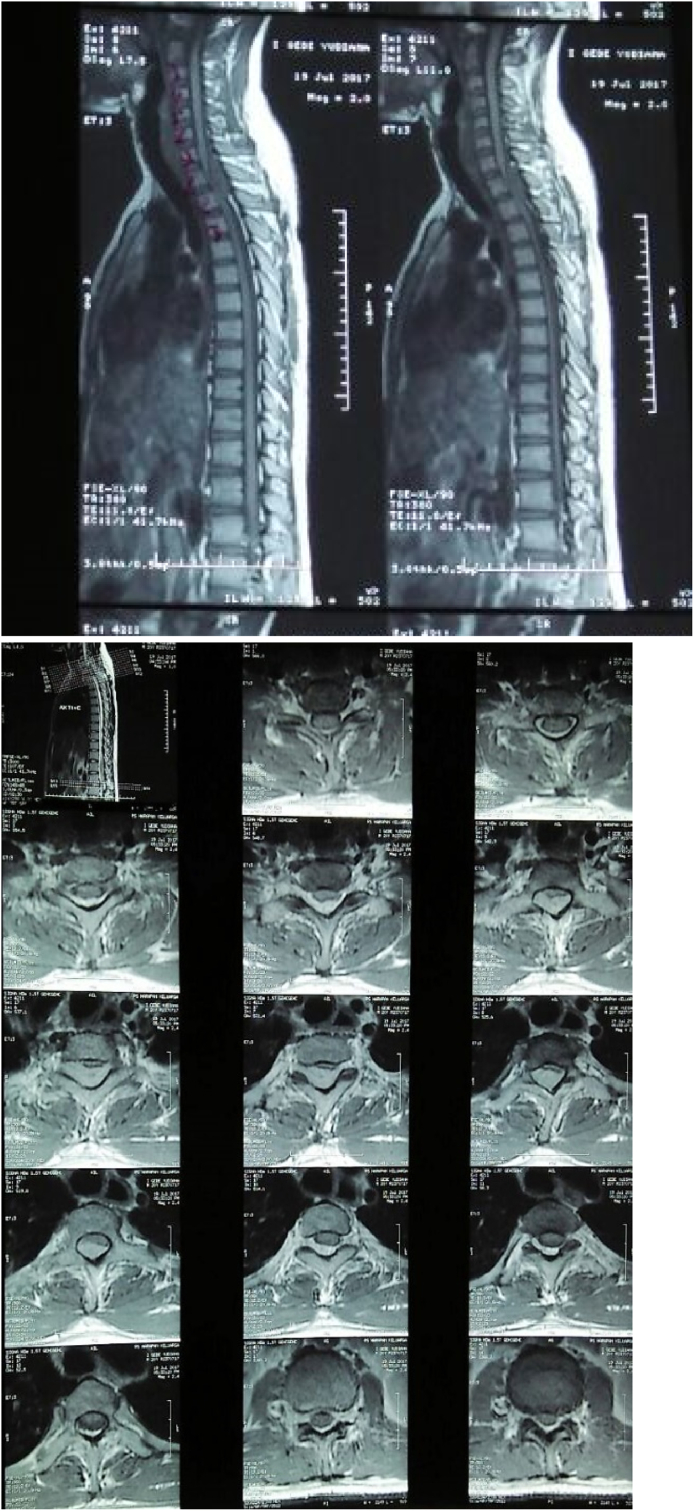
Magnetic Resonance (MR) images (T1 Sagital and Axial View with contrast) of the spine show an extradural mass in Th1–Th2. This image shows a heterogeneous thoracal mass corresponding to a schwannoma. Magnetic Resonance (MR) images of the Th1–Th2 spine show the tumor compressing the thoracal cord.

**Fig. 3:**
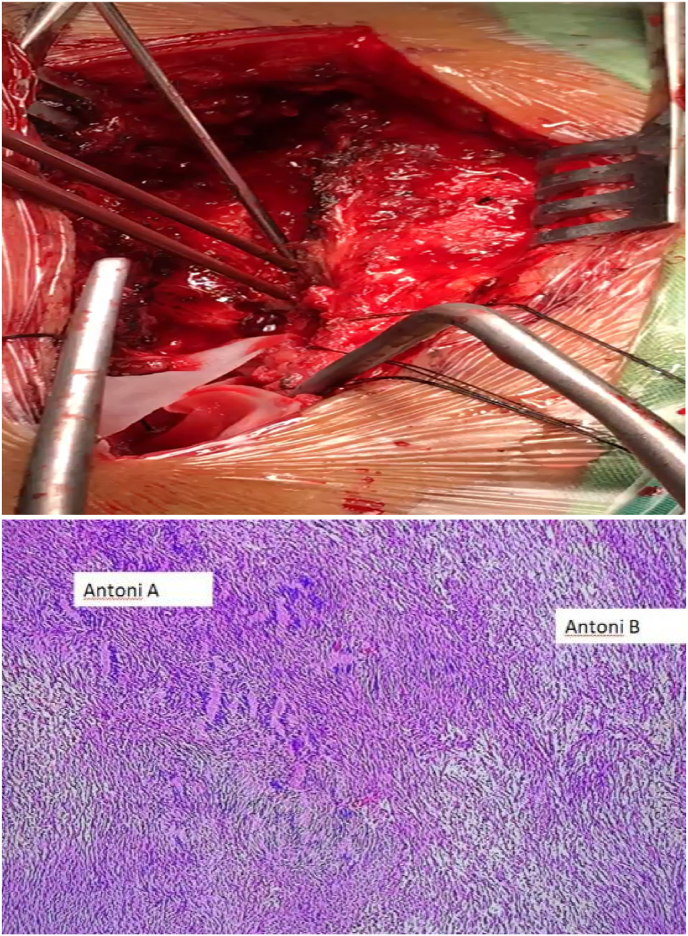
**a.** Hemilaminectomy and complete surgical resection. Intraoperative photograph showing the extradural tumor. **b.** Histopathological examination showed polymorphic spindle cells with area hypercellular and hypocellular, without malignancy sign.

His laboratory and thorax X-ray showed no apparent abnormalities as well. In imaging contrast, an MRI of the spine showed tumor extradural mass lesion extending from thoracal 1–2 vertebral level (Fig. 2). The patient underwent hemilaminectomy and complete surgical resection. Histopathological examination showed polymorphic spindle cells with area hypercellular and hypocellular, without malignancy signs. The final opinion was compatible with schwannoma (Fig. 3).

**Fig. 4:**
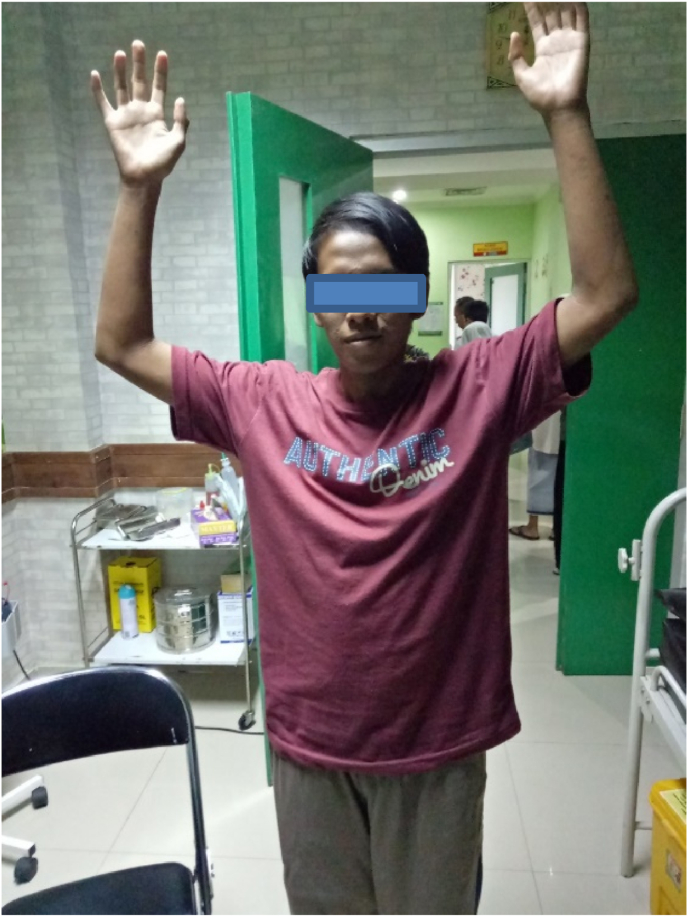
Evaluation patient 3 months after surgery the patient has no neurological symptoms. Before Surgery patient with spastic.

We diagnose the patient from clinical findings and imaging methods and MRI is the best method for diagnosis and differential diagnosis. The size and specific margins of the mass demonstrate the localization and invasion of the contiguous structures. We treat all patients with hemilaminectomy and complete surgical resection, after follow-up 3 months after surgery, the patient becomes normal from paraparesis and spastic (Fig. 4).

In these two patients, we only performed a hemilaminectomy and excision of the tumor with a guiding microscope, we did not install a pedicle screw because it was unnecessary and too excessive in our opinion, in principle, according to the surgical procedure, we could reach the tumor without destroying the stability of the spine. in both patients we used a guiding microscope without IOM (intra operative monitoring), our center is rural area, we don't have IOM but we try to give the best for patients.

## 3 Discussion

Schwannoma is a common nerve sheath tumor that usually arises from sensory nerves and is benign [[10], [11], [12]]. Some classifications are usually used in spinal schwannomas based on anatomical location. The first is intradural tumors (75%), which are divided into extramedullary, which are located within the dura but not part of the spinal cord. And intramedullary which is located in the parenchyma of the spinal cord. The second one is an extradural tumor (15%), which is present between the bone structure and the dura. The last one is intra-extradural (10%) [4,13,14]. according to the literature, there is no difference in prevalence predilection of schwannoma between males and females [11,15,16]. But some studies showed that Schwannoma is more prevalent in women [9].

In this case, the main complaint is paraparesis and followed by suffered low back pain and spastic. The initial symptoms of this tumor depend on the level of the tumor location. Usually begins with localized pain, sharp and transient, followed by radicular pain and radiculopathy. When the compression of the tumor in the spinal cord increases, will lead to damage to the spinal cord and causes myelopathy [12]. Numbness, and paraparesis may also appear, but in the present study, the main complaint was pain (79%) [17]. Motor deficit and sphincter disturbances are uncommon onset symptoms [18]. According to *Alqroom, R.* et al. the most common initial symptom was radicular pain (67%) followed by motor weakness, another 31% had myelopathy and sphincter disturbances [12]. Some studies had shown that deficit neurological should be expected following surgical treatment of these tumors [23].

In some cases, the location of schwannoma was more common in the cervical region (frequently in the high region, i.e. C1–C3 levels) and lumbar than thoracal [14,[17], [18], [19], [20], [21], [22]]. In the study of Alqroom, R. et al. showed that the higher incidence of the regions involved was the lumbar spine (55.22%), followed by cervical (23.88%), thoracic segments (17.91%), and sacral (2.98%) [12].

We reported cases of schwannoma of the thoracal spine that have presented with neurological involvement. MRI of the spine showed tumor extradural mass lesion extending from thoracal 1–2 vertebral and thoracal 4–6 vertebral level. The location of this tumor is rare because located in the extradural (15%), which are present between the bone structure and the dura. Magnetic resonance imaging (MRI) is the gold standard to diagnose especially in spinal tumors [24]. In MRI, we can determine details of the tumor and surrounding the structures and neural elements [25]. It showed Iso- or Hypo-intense on T1 weighted and hyperintense on T2 weighted images [26]. Meanwhile, X-Ray and Computed Tomography (CT) scans are used to evaluate bone windows i.e. details of bone erosion or destruction that can develop into spinal instability [25]. In this case, there were no apparent abnormalities as well in the thorax X-ray. So it can be concluded that there is no damage caused by the tumor.

According to *Sridhar* et al. suggested a classification of benign Spinal Schwannoma in various types. Type I-intraspinal tumor, <2 vertebral segmented in length; a:intradural; b: extradural. Type II-intraspinal tumor, >2 vertebral segments in length (giant tumor). Type III-intraspinal tumor with extension into nerve root foramen. Type IV-intraspinal tumor with extraspinal extension (dumbbell tumors); a: extraspinal component <2.5 cm; b: extraspinal component >2.5 cm (giant tumor). Type V-tumor with erosion into vertebral bodies (giant invasive tumor), lateral and posterior extensions into myofascial planes [[27], [28], [29]]. And *Park* et al. modified the classification by adding two new classifications. Type VI-tumor in an entirely intravertebral location without intraspinal portion. And type VI-intraspinal tumor with erosion into vertebral bodies and extension into nerve root foramen [28]. Therefore, this patient is classified as type II because the intraspinal tumor affects more than two spinal segments.

On histopathological examination, showed polymorphic spindle cells with area hypercellular and hypocellular, without malignancy sign. Spinal schwannoma has two types in histopathological findings, Antoni A (hypercellular) and Antoni B patterns (hypocellular) [4,30]. According to examination these patient types are both Antoni A and Antoni B because there are areas hypercellular and hypocellular.

Surgery is the treatment of choice in this case, which usually result from excellent prognostic [12]. This patient underwent hemilaminectomy and complete surgical resection. The parameters are based on tumor location, bone involvement, and symptoms [30]. The study by Abbasi et al. explained that patients with symptoms of impairment of their bladder and bowel do not significantly recover after surgical management [13]. Complete surgical resection was chosen in this case, Yüzbaşi et al. said early diagnostic and complete surgical resection before the occurrence of severe symptoms will show an excellent prognosis [14]. The writing of this script follows the rules of the SCARE 2020 Guideline. The work has been reported in line with the SCARE 2020 [31].

## 4 Conclusions

Spinal extradural schwannoma is a rare case, especially in thoracal. Early diagnostic and complete surgical resection should be the goal treatment, these also will significantly show good prognostic.

## Ethical approval

Obtained.

## Sources of funding

No funding or sponsorship.

## Author contribution

RHA, BAM, NUW, WYD, and KVG wrote the abstract, introduction, case, discussion, conclusion. RHA, BAM, NUW, WYD, and KVG performed critical edits and final revision, figures.

## Registration of research studies

1.Name of the registry: NA.

2.Unique Identifying number or registration ID: NA.

3.Hyperlink to your specific registration (must be publicly accessible and will be checked): NA.

## Guarantor

Rohadi Muhammad Rosyidi.

## Disclosure

The authors report no conflict of interest concerning the materials or methods used in this study or findings specified in this paper.

## Provenance and peer review

Not commissioned, externally peer-reviewed.

Written informed consent was obtained from the patient for publication of this case report and accompanying images. A copy of the written consent is available for review by the Editor-in-Chief of this journal on request.

## Declaration of competing interest

None.
